# Glucagon Regulation of Energy Expenditure

**DOI:** 10.3390/ijms20215407

**Published:** 2019-10-30

**Authors:** Maximilian Kleinert, Stephan Sachs, Kirk M. Habegger, Susanna M. Hofmann, Timo D. Müller

**Affiliations:** 1 Institute for Diabetes and Obesity, Helmholtz Diabetes Center at Helmholtz Centre Munich, Ingolstädter Landstraße 1, 85764 Oberschleißheim, Germany; maximilian.kleinert@helmholtz-muenchen.de (M.K.);; 2 Section of Molecular Physiology, Department of Nutrition, Exercise and Sports, University of Copenhagen, 2100 Copenhagen, Denmark; 3 Division of Metabolic Diseases, Technische Universität München, 85740 Munich, Germany; 4 Department of Medicine-Endocrinology and Comprehensive Diabetes Center, Diabetes and Metabolism, University of Alabama at Birmingham, Birmingham, AL 35899, USA; 5 Institute for Diabetes and Regeneration, Helmholtz Diabetes Center at Helmholtz Zentrum München, German Research Center for Environmental Health (GmbH), 85764 Neuherberg, Germany; 6 German Center for Diabetes Research (DZD), 85764 Neuherberg, Germany; 7 Medizinische Klinik und Poliklinik IV, Klinikum der LMU, 80336 München, Germany; 8 Department of Pharmacology and Experimental Therapy, Institute of Experimental and Clinical Pharmacology and Toxicology, Eberhard Karls University Hospitals and Clinics, 72076 Tübingen, Germany

**Keywords:** glucagon, obesity, energy expenditure, FGF21, brown adipose tissue, pharmacology

## Abstract

Glucagon’s ability to increase energy expenditure has been known for more than 60 years, yet the mechanisms underlining glucagon’s thermogenic effect still remain largely elusive. Over the last years, significant efforts were directed to unravel the physiological and cellular underpinnings of how glucagon regulates energy expenditure. In this review, we summarize the current knowledge on how glucagon regulates systems metabolism with a special emphasis on its acute and chronic thermogenic effects.

## 1. Introduction

Glucagon is a 29 amino acid peptide that is secreted from pancreatic α-cells in response to low levels of blood glucose. Circulating glucagon levels are thus low in the prandial state (~10 pmol/L in humans) and increase 2–3 fold in response to long-term fasting or hypoglycemia. Glucagon acts through the glucagon receptor (GCGR), a G protein-coupled receptor that is most abundantly expressed in the liver. Traces of GCGR can also be found in several extrahepatic tissues, such as the brain, heart, kidney, gastrointestinal tract and adipose tissues [1].

The best-described effect of glucagon is its action on the liver, where it increases blood glucose through stimulation of gluconeogenesis and glycogenolysis [2]. Glucagon’s rapid effect to stimulate hepatic glucose production renders glucagon a valuable emergency medication for the treatment of acute severe hypoglycemia [3]. Nonetheless, glucagon has gained little pharmacological attention outside its role to rescue hypoglycemia. Often reduced to its glycemic effects, glucagon is a pleiotropic hormone with metabolic action that goes well beyond its ability to increase blood glucose. These non-glycemic effects of glucagon include the modulation of food intake and satiety [4], lipid homeostasis [5], insulin secretion [6], and energy expenditure [7].

Obesity is the consequence of a persistent positive imbalance between energy intake and expenditure. Metabolizable energy depends upon both energy intake as well as the efficacy by which nutrients are absorbed in the intestine. To maintain a stable bodyweight, the energy made available for metabolism needs to make up for the bodies energetic demand for growth, maintenance (basal metabolic rate), physical activity, pregnancy and lactation, and other factors. Ideally, interventions to treat obesity target both, energy intake and energy expenditure. Intriguingly, glucagon has been shown to promote satiety and to increase energy expenditure in both rodents and humans. The satiety effect of glucagon is likely mediated via the liver-vagus-hypothalamus axis, since disconnection of the hepatic branch of the abdominal vagus nerve blocks glucagon’s anorectic effect [8]. Plasma glucagon levels increase after the ingestion of a mixed meal [9,10,11] and pre-prandial injection of antibodies against glucagon increased food intake [12], indicating a physiological relevance for glucagon in regulation of satiety.

However, the pharmacological value of glucagon to treat obesity is nonetheless hampered by glucagon’s hyperglycemic nature. When used as a stand-alone therapy, glucagon can contribute to the development of hyperglycemia. In line with this notion, postprandial hyperglucagonemia has been associated with hyperglycemia and the development of type-2 diabetes [13]. A series of preclinical [14,15,16,17] and clinical studies [18,19] have demonstrated that inhibition of glucagon signaling can have beneficial effects on glucose metabolism in individuals with type-2 diabetes. However adverse effects such as increased hepatic fat accumulation and increased LDL cholesterol have been reported for these antagonists in humans [20,21].

The non-glycemic effects of glucagon render this molecule an interesting target for the treatment of obesity. A better understanding of how glucagon regulates energy expenditure might, therefore, offer the possibility to pharmacologically accentuate glucagon’s thermogenic effect without detrimental effects on blood glucose. In this review, we summarize the current knowledge on how glucagon regulates energy expenditure, with a special emphasize on acute and chronic effects.

## 2. The Effect Size of Glucagon-Induced Energy Expenditure in Humans

The ability of glucagon to increase energy expenditure was first demonstrated in rats in 1960 [22] and was subsequently confirmed in humans [23]. Glucagon-stimulation of energy expenditure has since then been shown in a series of human studies (Table 1), albeit with some exception [24]. In most human studies, the thermogenic effect of glucagon is rapid, with elevated oxygen consumption observed within minutes of infusion into the general circulation.

In one of the first human studies to assess glucagon-mediated changes in energy expenditure, glucagon was infused in overnight fasted subjects at a rate of 3 ng/kg/min, resulting in a 5-fold increase in circulating glucagon and an increase in energy expenditure of 240 kcal/day [23]. In this study, somatostatin was co-infused to suppress the endogenous release of insulin and glucagon from the pancreas [23]. In a more recent human study, glucagon was infused at a rate of 50 ng/kg/min, resulting in a 25-fold increase in circulating glucagon and an increase in energy expenditure of 150 kcal/day [25]. Somatostatin was not co-infused in this study and subjects had consumed a light standardized breakfast two hours prior to the initiation of treatment [25]. In another study in overnight fasted subjects, the same dose of glucagon (50 ng/kg/min) increased energy expenditure by 230 kcal/day [26]. These data suggest that glucagon-stimulation of energy expenditure varies substantially between studies and indicates that the individuals’ feeding status (pre-prandial vs. post-prandial) is an important factor when analyzing glucagon effects on energy expenditure. Consistent with this line of reasoning, glucagon (3 ng/kg/min) failed to affect energy expenditure when infused directly after a meal [24]. Furthermore, glucagon-induction of energy expenditure is suppressed when glucagon is co-infused with high concentrations of insulin [27]. While glucagon effects on thermogenesis are markedly suppressed in the prandial state, intranasal administration of as little as 0.7 mg glucagon is sufficient to increase energy expenditure by 207 kcal/day in overnight fasted subjects, despite only a transient 2-fold increase in circulating glucagon [28]. In summary, a series of human studies confirmed the acute thermogenic effect of glucagon and glucagon stimulation of energy expenditure seemingly depends on the feeding status with diminished ability of glucagon to stimulate energy expenditure in the prandial state and elevation of energy expenditure by about 100–200 kcal when infused during the pre-prandial state. Glucagon-induced energy expenditure is preserved in human subjects with obesity [25,28]. The magnitude of glucagon’s thermogenic effect is similar to that of the ß3-adrenergic receptor agonist mirabegron, which primarily targets the brown adipose tissue (BAT) (+203 kcal/day) [29] and is similar to the acute energy expenditure increase induced by cold exposure (+193 kcal/day) [28]. Consequentially, it might be hypothesized that glucagon carries a sizable pharmacological potential to decrease bodyweight due to its thermogenic and anorectic effects.

## 3. Glucagon-Induced Energy Expenditure: Acute vs. Chronic Effects

Nearly 60 years ago, it was described that rats treated chronically with daily glucagon gain substantially less bodyweight and fat mass as compared to pair-fed rats [22,30]. Since then, the acute thermogenic effect of glucagon has been confirmed in various species including humans (Table 1), rodents [31], pigs [32], and dogs [33].

While the ability of glucagon to also chronically induce energy expenditure has repeatedly been demonstrated in rodents [34,35], studies of chronic or even subchronic glucagon administration in humans are scarce. Regarding glucagon’s thermogenic effect, it is yet still unclear (1) how and at what anatomic localizations glucagon act to increase energy expenditure, (2) whether different signaling mechanisms account for the acute and chronic thermogenic effect of glucagon and (3) whether glucagon’s acute and chronic thermogenic effects show species-related differences.

## 4. Mediators of the Acute Effect of Glucagon on Energy Expenditure

### 4.1. Role of the Brown Adipose Tissue

In small rodents and hibernators, the BAT can account for up to 50% of basal metabolic rate [36]. While BAT thermogenesis is now established as a relevant therapeutic target in adult humans [37], it must be noted that the overall contribution of BAT thermogenesis on whole body energy homeostasis is far more important for rodents relative to humans. Considering these species-related differences when deciphering glucagon’s thermogenic effect is of particular importance since mice as compared to humans are particularly prone to show energy expenditure differences upon BAT stimulation.

It was already noted in 1966 that glucagon increases oxygen consumption in isolated BAT cells and BAT tissue explants of rats [38,39], indicating that glucagon acts on BAT autonomously to stimulate energy expenditure. Notably, most *in vitro* studies assessing glucagon effects on BAT function used supra-physiological doses, which limit the conclusions for its role in regulating thermogenesis under physiological conditions [40]. Nonetheless, glucagon has been demonstrated to increase the temperature over interscapular BAT in newborn rabbits [41,42]. In rats, blood flow into BAT increases following glucagon administration [43], which is in accordance to glucagon’s effect on vasodilation [44] and cardiac output [45,46]. Glucagon’s effect on energy expenditure is also potentiated in mice that are adapted to cold, an experimental condition in which the mass of BAT and its capacity for heat production is increased [47].

**Figure 1:**
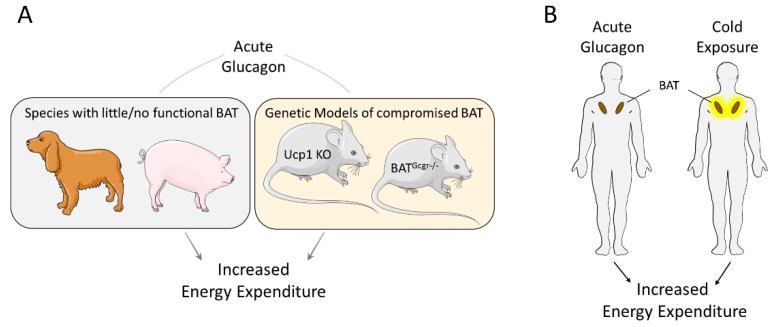
Acute effects of glucagon are not solely mediated by brown adipose tissue (BAT). (**A**) Acute administration of glucagon increases energy expenditure in animal species with little or no functional BAT, like dogs and pigs, and in genetically modified mice lacking functional Ucp1 gene (Ucp1 KO) or glucagon receptor in brown adipose tissue (BAT^Gcgr−/−^). (**B**) Both cold exposure and glucagon administration increased energy expenditure to a similar extent, while only cold exposure increased BAT activity.

These studies above suggest that acute glucagon administration affects energy expenditure via BAT. However, there is also considerable literature indicating that glucagon affects thermogenesis via BAT-independent mechanisms, including studies in species with little BAT (adult dogs) or no BAT (pigs) activity. In both of these species, glucagon acutely increases energy expenditure [32,33]. BAT thermogenesis relies largely on uncoupling protein 1 (UCP1) located in the inner membrane of mitochondria. UCP1 uncouples oxidative phosphorylation from ATP synthesis with the result that energy form substrate oxidation is released as heat [48,49]. In mice lacking UCP1, glucagon increases energy expenditure without difference compared to wildtype controls [31]. Consistent with this, mice with selective ablation of the glucagon receptor in BAT increase their energy expenditure to the same extent as wildtype mice following glucagon injection [31]. Collectively, this suggests that *in vivo* neither BAT nor glucagon receptor signaling in BAT are required for the acute energy expenditure effect of glucagon in mice (Figure 1). It is unclear if this conclusion also applies to rats. BAT cells derived from rats are roughly 200-fold more sensitive to glucagon stimulation of oxygen consumption relative to BAT cells isolated from mice [40]. Whether this is due to differences in BAT glucagon receptor abundance is unknown, but it raises the possibility that glucagon has a greater physiological role to stimulate BAT thermogenesis in rats relative to mice. However, as assessed by mitochondrial GDP binding, BAT activity increases only after multiple days of glucagon treatment, and is not altered 2 h after the first injection, a time point at which energy expenditure is already increased [50]. This argues that glucagon also affects acute energy expenditure in the rat via BAT-independent mechanisms (Figure 1).

In humans, the question whether glucagon acutely activates BAT was recently investigated in eight young men selected for active BAT, as determined by 18F-fluorodeoxyglucose positron emission tomography/CT (18F-FDG PET/CT) during a cold challenge. In these BAT-positive subjects, glucagon infusion (50 ng/kg/min for 55 min) increased energy expenditure by 230 kcal/day without an observed increase in BAT activity [26]. Collectively, these data further corroborate that glucagon effects on energy expenditure are BAT independent (Figure 1).

### 4.2. Potential Mechanisms for Acute Glucagon-Induced Energy Expenditure

Glucagon is well known to elevate the circulating pool of glucose via its hepatic actions [51]. In addition, glucagon also promotes synthesis of ketone bodies from hepatic fatty acid oxidation [52]. Thus, glucagon has system-wide effects, leading, for instance, to augmented release of amino acids from peripheral organs and a simultaneous increased oxidation of amino acids in the liver [53]. Similarly, glucagon is thought to stimulate lipolysis in white adipose tissue [54,55], plausibly providing energy substrates for the liver and other organs such as BAT. The metabolic cost of glucagon’s catabolic effects together with the subsequent counter-regulatory anabolic response to re-store these metabolites could create a futile cycle contributing to the increase in energy expenditure. This is difficult to formally test, because it may comprise small (i.e., difficult to measure) changes in different aspects of energy substrate turnover, yet the sum of these changes might be meaningful. A diet switch to a high protein carbohydrate-free diet highlights the potential metabolic cost of gluconeogenesis, as this increases oxygen consumption in humans after one day, with hepatic gluconeogenesis contributing to 40% to this rise in energy expenditure [56]. Interestingly, acute glucagon administration directly increases liver energy expenditure [57,58,59] and glucagon treatment has been shown to increase oxidative phosphorylation in isolated liver mitochondria [60,61,62]. A model in which various catabolic effects of glucagon contribute to increased energy expenditure may explain why insulin negatively impacts glucagon’s thermogenic effect. Generally, somatostatin-suppression of insulin secretion enhances the thermogenic effect of glucagon, while glucagon fails to affect energy expenditure upon co-infusion of high-dose insulin [27] (Table 1).

It should also be noted that glucagon increases circulating levels of cortisol in humans [63]. It was already demonstrated in 1960 that glucagon fails to increase the metabolic rate of adrenalectomized rats, which decreases endogenous catecholamines and cortisol [22]. Treating adrenalectomized rats with exogenous cortisol fully restores glucagon’s ability to acutely increase EE, suggesting that glucagon also affects energy expenditure via the hypothalamus-pituitary-adrenal (HPA) axis and thus actions on the sympathetic nervous system (SNS) [22]. In line with this notion, acute infusion of cortisol increases energy expenditure in humans [64,65,66].

In summary, glucagon administration engages multiple metabolic pathways and it is plausible that the sum of these independent catabolic actions contributes to the increased energy expenditure observed with acute glucagon. A potential downside to eliciting these catabolic effects with glucagon may be that chronically they could trigger peripheral tissue wasting [67].

## 5. Mediators of the Chronic Effect of Glucagon on Energy Expenditure

The pioneering pair-feeding study of Salter in 1960 indicated that the chronic GcgR agonism regulates bodyweight via both food intake dependent and independent mechanisms [30]. Similarly, in obese Zucker rats, chronic glucagon administration limits bodyweight gain without affecting food intake [68]. Twice daily injections with glucagon at a dose of 1 mg/kg (~287 nmol/kg) for up to 18 days attenuated weight gain and increased BAT mass, BAT protein, and BAT DNA content in rats [69]. Chronic glucagon also increased mitochondrial GDP binding, a proxy of BAT thermogenic activity [69].

Despite a series of data emphasizing the beneficial effects of glucagon on bodyweight, its pharmacological potential has been limited by fears of adverse glycemic effects. However, in 2009, it was demonstrated that glucagon’s hyperglycemic effects can be balanced by the anti-hyperglycemic properties of glucagon-like peptide-1 (GLP-1), as demonstrated by a monomeric peptide with balanced activity at the GLP-1 and glucagon receptors [70,71]. These peptide co-agonists corrected adiposity, hepatic steatosis, hypercholesterolemia, and glucose intolerance in DIO rodents and several of such peptide hybrids are currently in clinical evaluation [70,71,72,73,74]. Notably, part of the metabolic benefits residing in GLP-1 and glucagon dual-pharmacology comprise the energy expenditure effects of glucagon, shown by the increase of energy expenditure in mice treated with GLP-1/glucagon but not with GLP-1 alone and diminished metabolic effect in mice deficient for the GLP-1 receptor [70].

In 2013, a long-acting, water-soluble, and highly selective glucagon receptor agonist (acyl-glucagon; IUB288) was developed [34]. The half-life (t_1/2_) of acyl-glucagon is ~63 times longer compared to naïve glucagon (t_1/2_~5–6 min) [34]. In diet-induced obese (DIO) C57B6/J mice, chronic administration of once daily acyl-glucagon at a dose of 10 nmol/kg over 18 days substantially reduced body weight (up to 25%) and increased energy expenditure [34,35]. In comparison, Billington et al. injected naïve glucagon at a dose of ~287 nmol/kg twice daily to observe an attenuation in weight gain in rats [69].

**Figure 2:**
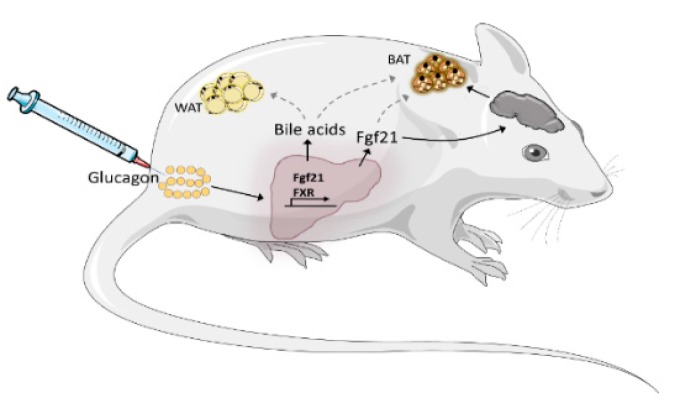
Proposed model of how chronic glucagon treatment increases energy expenditure in mice. Chronic glucagon treatment increases the synthesis and secretion of hepatic Fgf21. Fgf21, in turn, augments the sympathetic output and innervation of BAT via actions on the brain, increasing energy expenditure. Whether chronic glucagon has direct metabolic effects on WAT and BAT is uncertain. In addition, chronic glucagon treatment increases circulating bile acids, which are known modulators of whole body energy metabolism. To which extent bile acids mediate the chronic effects of chronic glucagon treatment is under current investigation.

Notably, glucagon fails to promote body weight loss in mice that specifically lack the glucagon receptor in liver [35]. Expression of GcgR is by far greatest in liver, with levels being 1,000-fold higher compared to GcgR expression in BAT [31,75]. In the liver, glucagon stimulates the synthesis and release of fibroblast growth factor 21 (FGF21) [34,76], a circulating peptide hormone that regulates energy homeostasis [77,78] and is primarily derived from the liver [35,79] (Figure 2). Fgf21 stimulates the thermogenic gene expression in isolated brown (and white) fat cells of mice [80]. However, *in vivo* these direct effects are dispensable, as the adipocyte-specific deletion of the Fgf21 receptor (Klb) had no effect on Fgf21′s ability to reduce bodyweight in DIO mice [80]. This suggests that Fgf21 increases energy expenditure via centrally mediated mechanisms [81,82] (Figure 2). Chronic GcgR agonism fails to increase energy expenditure and prevents HFD-induced obesity in Fgf21 null mice [34]. In obese liver-specific Fgf21-deficient mice, chronic glucagon-mediated bodyweight loss is blunted [35], without effects on food intake. These studies suggest that hepatic Fgf21 secretion contributes to the chronic effects of glucagon on energy expenditure (Figure 2).

In addition to Fgf21, chronic glucagon treatment increases circulating levels of bile acids in DIO mice [35]. Bile acids are ligands for the farnesoid X receptor (FXR) [35] and both, bile acids and FXR, regulate energy expenditure [83]. In liver-specific FXR knockout mice, the bodyweight lowering effects of glucagon is blunted, despite normal Fgf21 secretion [35]; suggesting that in addition to Fgf21, a hepatic bile acid-FXR axis contributes to the chronic effects of glucagon on energy expenditure (Figure 2).

Clearly, the liver is pivotal in mediating glucagon’s anti-obesity effects, but the contribution of other organs remains to be determined. Davidson and colleagues found that an acute injection of glucagon in adrenalectomized and thyroidectomized rats did not increase energy expenditure [22]. Chronic treatment of DIO mice with the acyl-glucagon increased circulating T3 and T4 levels [35]. Together, these results suggest that other secreted hormones (e.g., epinephrine, cortisol (both adrenal gland) and thyroid hormone) play a role in glucagon’s thermogenic effect.

Glucagon can cross the blood brain barrier [84] and the GcgR is expressed in hypothalamus and brainstem regions, two sites known to modulate energy metabolism [85,86]. Single intracerebroventicular (ICV) infusion of glucagon increases energy expenditure in rodents [87,88], and this is accompanied by stimulation of BAT thermogenesis [88]. Chronic ICV studies assessing the role of glucagon on energy expenditure have, to the best of our knowledge, not been performed. Interestingly, central glucagon signaling also appears to play an important role in regulating hepatic glucose production [89,90,91]. Whether distinct or overlapping central pathways regulate both energy expenditure and glycaemia is unknown.

The GcgR is also expressed in the white adipose tissue (WAT) of rodents (~100× higher than in BAT) and humans [5]. Whether chronic GcgR activation in the WAT contributes to the thermogenic effects of glucagon action is unknown, but lipolytic effects might be involved [92].

Taken together, studies in genetically modified mice indicate that GcgR agonism at the liver, with the stimulation of bile acid and Fgf21 secretion, is the primary mediator of glucagon’s effect on energy metabolism (Figure 2). The role of these pathways in humans and the importance of other organs remain to be elucidated.

## 6. Conclusions

Glucagon regulation of energy metabolism is a complex process that likely involves bi-directional cross-talk between key peripheral organs and the brain. The liver as the primary expression site of the glucagon receptor seems to play a pivotal role in regulating glucagon’s chronic thermogenic effects. Importantly, glucagon regulation of energy expenditure might underlie species-specific differences and different mechanisms might contribute to glucagon’s acute and chronic effects on energy expenditure. Moreover, the acute effects of glucagon to increase oxygen consumption are not fully dependent on functional brown adipose tissue. Nonetheless, when given chronically, glucagon appears to increase the thermogenic capacity of brown adipose tissue via liver-specific mechanisms that among others include signaling of FGF21 and bile acids.

## Figures and Tables

**Table 1:** Studies assessing glucagon’s effect on energy expenditure in humans.

Glucagon Administration	Increase in Circulating Glucagon	Co-Infusion	Prandial State	Delta EE (kcal/day)	Ref.
6 ng/kg/min; infused	3.5-fold	Somatostatin; Insulin (0.15 mU/kg/min)	Overnight fasted	+75	[22]
6 ng/kg/min; infused	3.5-fold	Somatostatin; Insulin (0.45 mU/kg/min)	Overnight fasted	No effect	[22]
3 ng/kg/min; infused	5-fold	Somatostatin	Overnight fasted	+240	[18]
3 ng/kg/min; infused	5–6-fold	--	Meal right before infusion	No effect	[19]
0.7 mg; intranasal	Transient 2-fold	--	Overnight fasted	+207	[23]
50 ng/kg/min; infused	25-fold	--	Meal 2 h before infusion	+150	[20]
50 ng/kg/min; infused	not shown	--	Overnight fasted	+230	[21]
